# Supplementary material for: Expression of USP25 associates with fibrosis, inflammation and metabolism changes in IgG4-related disease
Source: Nat Commun. 2024 Mar 23;15:2627. doi: 10.1038/s41467-024-45977-7 (PMC10960850; doi:10.1038/s41467-024-45977-7)
Supplement: Supplementary file 1 — Supplementary Information [file 41467_2024_45977_MOESM1_ESM.pdf]

Supplementary Information

Expression of USP25 associates with fibrosis, inflammation and metabolism changes in IgG4-related disease

Panpan Jiang[1], Yukai Jing[1], Siyu Zhao[2], Caini Lan[1], Lu Yang[1], Xin Dai[1], Li Luo[1], Shaozhe Cai[3], Yingzi Zhu[3], Heather Miller[4], Juan Lai[5], Xin Zhang[5], Xiaochao Zhao[5], Yonggui Wu[6], Jingzhi Yang[7], Wen Zhang[8], Fei Guan[1], Bo Zhong[9,10], Hisanori Umehara[11], Jiahui Lei[1], Lingli Dong[3]*, and Chaohong Liu[1]*

[1]Department of Pathogen Biology, School of Basic Medicine, Tongji Medical College and State Key Laboratory for Diagnosis and Treatment of Severe Zoonotic Infectious Diseases, Huazhong University of Science and Technology, Wuhan, 430030, Hubei, China.

[2]Department Immunology, School of Medicine, Yangtze University; Jingzhou 434000, China.

[3]Department of Rheumatology and Immunology, Tongji Hospital, Tongji Medical College, Huazhong University of Science and Technology; Wuhan 430000, China.

[4]Cytek Biosciences, R&D Clinical Reagents, Fremont, CA, United States.

[5]GeneMind Biosciences Company Limited; Shenzhen 518001, China.

[6]Department of Nephropathy, the First Affiliated Hospital of Anhui Medical University, Hefei, Anhui 230022, PR China; Center for Scientific Research of Anhui Medical University, Hefei, Anhui 230032, PR China.

[7]Department of Orthopedics, Qilu Hospital of Shandong University, Jinan, Shandong 250063, PR China.

[8]Department of Rheumatology, Peking Union Medical College Hospital, Chinese Academy of Medical Science & Peking Union Medical College, National Clinical Research Center for Dermatologic and Immunologic Diseases, State Key Laboratory of Complex Severe and Rare Diseases, Beijing 100730, China.

[9]Department of Gastrointestinal Surgery, Medical Research Institute, Frontier Science Center for Immunology and Metabolism, Zhongnan Hospital of Wuhan University, Wuhan University, Wuhan, 430071, China.

[10]TaiKang Center for Life and Medical Sciences, Wuhan University, Wuhan, 430071, China.

[11]Department of Medicine, Nagahama City Hospital; Nagahama 949-1701, Japan.

*Corresponding authors: tjhdongll@163.com (L.D.) and chaohongliu80@126.com (CH.L.)

Index: Supplementary figures S1 to S10 and Tables S2 to S5

Supplementary Figure 1

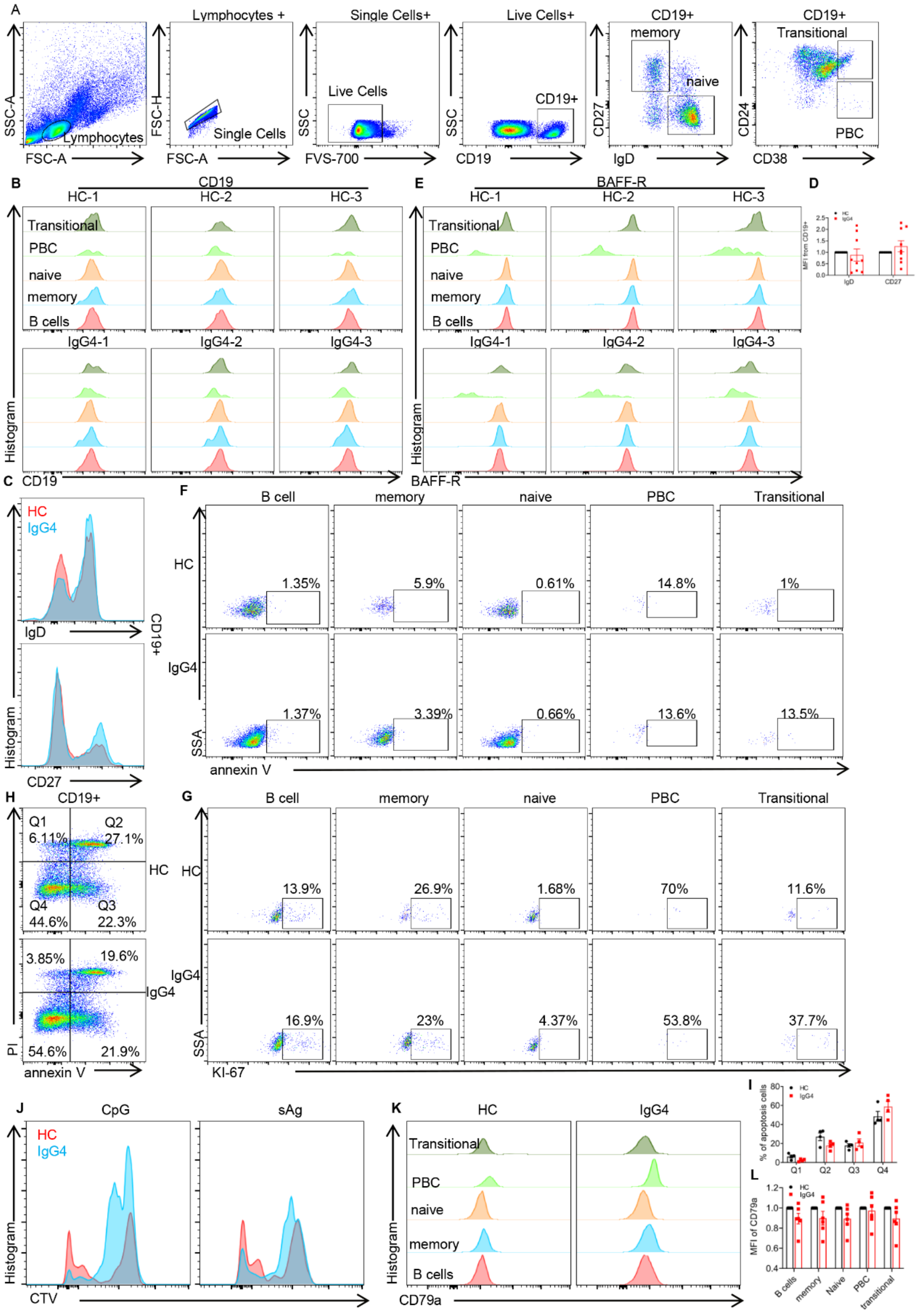

Supplementary Figure 1: IgG4-RD patients have altered B cell homeostasis.

(A) The overall gating strategies for flow cytometry data. (B) Analysis of the MFI of CD19 of the B cell subsets of PBMCs from IgG4-RD patients and HCs by flow cytometry. Shown are representative dot plots (n=3). (C-D) The MFI of IgD and CD27 in IgG4-RD patients and HCs CD19+ cells were analyzed by flow cytometry. Shown are representative plots (n=9). (E) Analysis of the MFI of BAFF-R of the subpopulations of PBMCs from IgG4-RD patients and HCs by flow cytometry. Shown are representative dot plots (n=3). (F-G) Analysis of the percentages of annexin V and KI-67 of the subpopulations of PBMCs from IgG4-RD patients and HCs by flow cytometry. Shown are representative dot plots. (H-I) PBMCs from HCs and IgG4-RD patients were stained with annexin V-FITC/PI and analyzed for the percentage of dead cells (Q1: Annexin $V^{-}PI^{+}$), late apoptotic cells (Q2: Annexin $V^{+}PI^{+}$), early apoptotic cells (Q3: Annexin $V^{+}PI^{-}$) and non-apoptotic cells (Q4: Annexin $V^{-}PI^{-}$) (n=5). (J) Purified B cell proliferation in HCs and IgG4-RD patients on day 3 after stimulation with CPG (10 μg/ml) and day 4 after stimulation with sAg (3 μg/ml) (n=3). (K-L) Analysis of the MFI of CD79α of the B cell subsets of PBMCs from IgG4-RD patients and HCs by flow cytometry. Shown are representative plots (n=6). All images were representative images from 3 independent experiments. Statistical significance was based on two-tailed unpaired Student's t-test. Relevant p values are given in the graph. *P < 0.05; **P < 0.01; ***P < 0.001. Source data are provided as a Source Data file.

Supplementary Figure 2

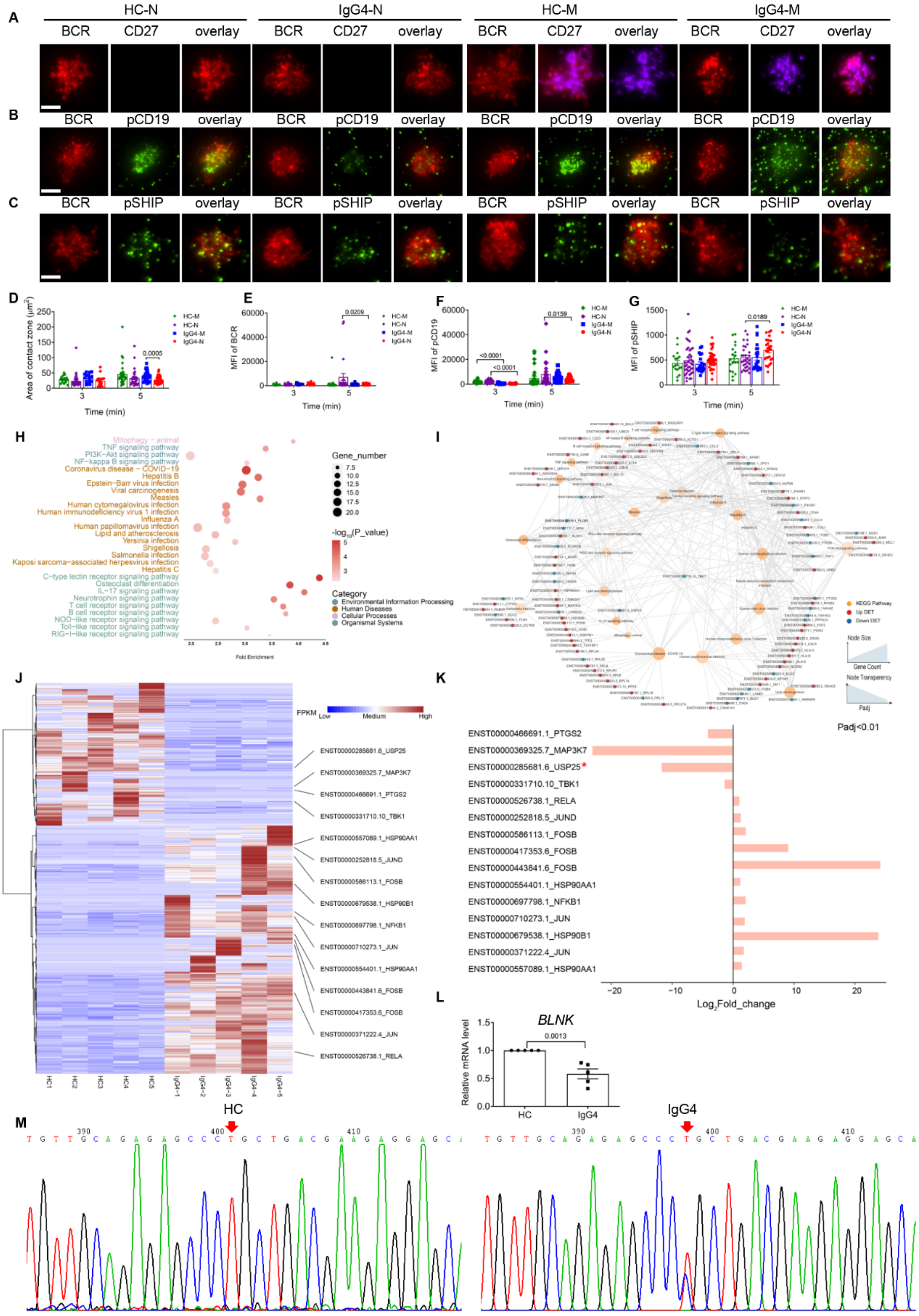

Supplementary Figure 2: IgG4-RD patients have immunodysregulation.

(A-G) Memory B (CD27+) cells and naive (CD27−) B cells of IgG4-RD patients and HCs were incubated in the same way as in (Fig.1I) except stained for AF-647 anti-CD27 (A), pCD19 (B) and pSHIP (C). Shown are representative images captured using TIRFm, and the B cells were analyzed for contact area (D), MFI of BCR (E), pCD19 (F), and pSHIP (G) (scale bar = 2.5 μm). (H) KEGG pathway enrichment analysis of DEGs in B cells of IgG4-RD patients and HCs. Enriched KEGG pathways (pvalue＜0.05) of DETs (|log2 fold change|≥1, p.adj＜0.01) (n=5). (I) The network of enriched KEGG pathways (pvalue＜0.05) of DETs (|log2 fold change|≥1, p.adj＜0.01) (n=5). (J) The heatmap shows that 611 (388 up-regulated, 223 down-regulated) significant DETs were identified between IgG4-RD and HC samples, DETs (|log2 fold change|≥1, p.adj＜0.01) (n=5). (K) Heatmap of genes involved in the IL-17 signaling pathway. ENST00000285681_USP25 transcript was the significant down-regulated in IgG4-RD patients (n=5). (L) Relative mRNA levels of *BLNK* in B cells were obtained from IgG4-RD patients and HCs (n=5). (M) Genomic DNA from HCs and IgG4-RD patients were PCR-amplified and analysis of mutation sites by direct bidirectional sequencing of PCR products (n=10). All images were representative images from 3 independent experiments. Statistical significance was based on two-tailed unpaired Student’s t-test. Relevant p values are given in the graph. *P < 0.05; **P < 0.01; ***P < 0.001. Source data are provided as a Source Data file.

Supplementary Figure 3

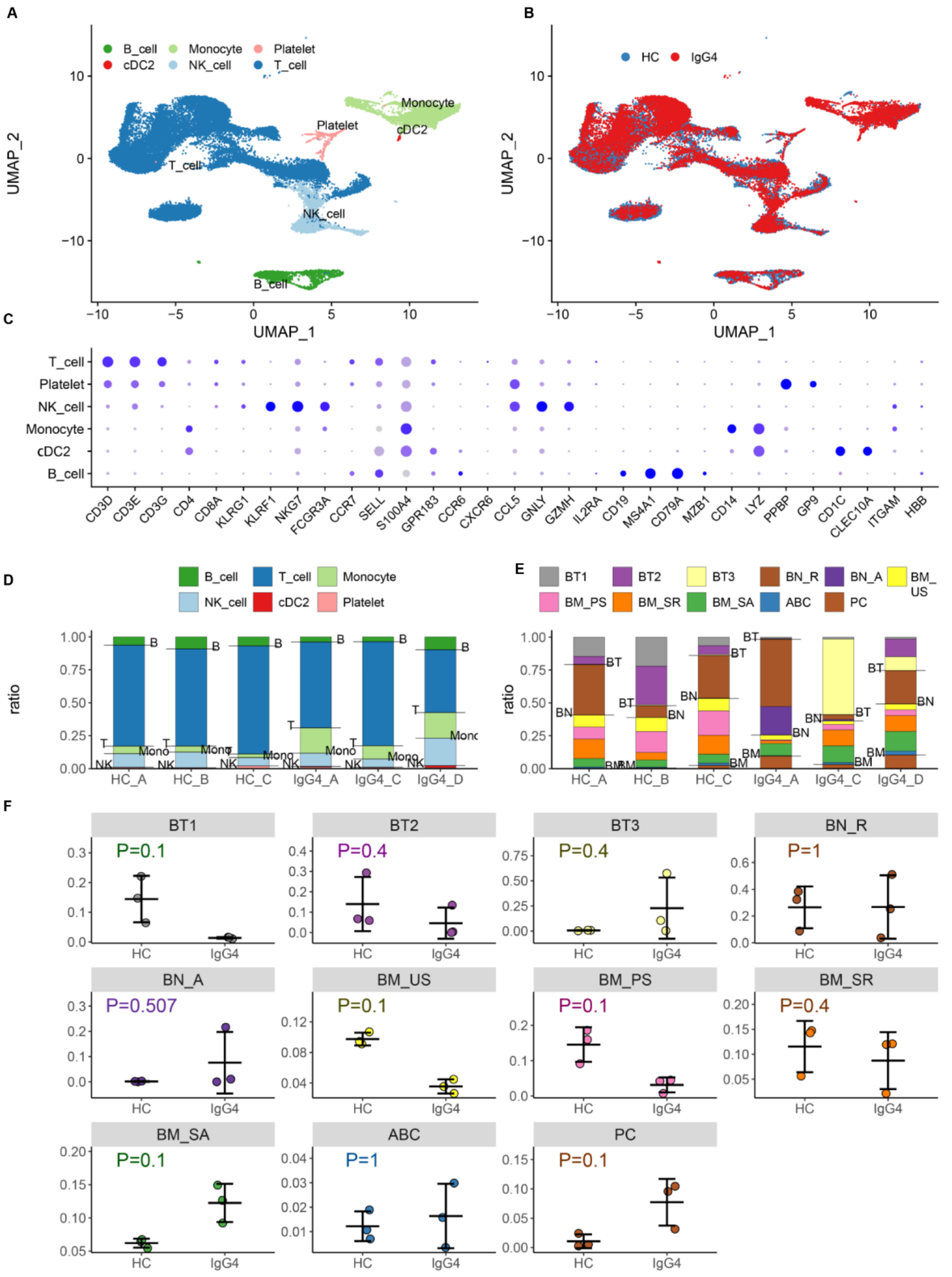

Supplementary Figure 3: Single-cell RNA-sequencing profiling of PBMCs in IgG4-RD.

(A-B) Integration analysis results, show UMAP formation of 6 main cell types (A) and status (B) (n=3). (C) Dot plot showing the expression of selected canonical cell markers in the 6 main cell types. (D-E) The relative abundance of immune cells in each sample from single-cell RNA-sequencing of total PBMCs (D) and B cells (E) (n=3). (F) The percentage of B cell subsets in total B cells from single-cell RNA-sequencing. Statistical significance was based on wilcoxon rank sum test. Relevant p values are given in the graph. *P < 0.05; **P < 0.01; ***P < 0.001. Source data are provided as a Source Data file.

Supplementary Figure 4

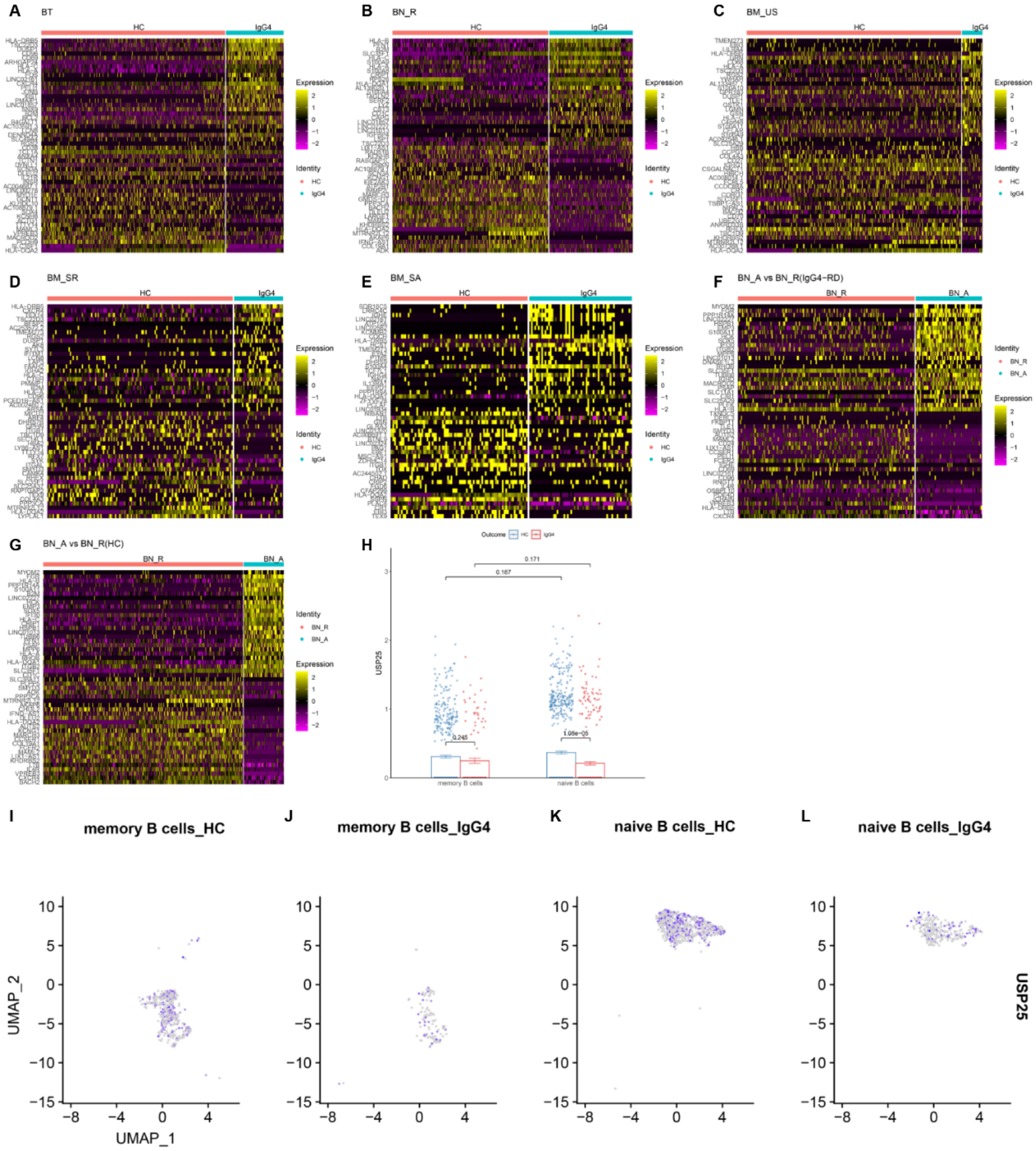

Supplementary Figure 4: Heat maps of DEGs in each B cell subset.

(A-E) Heat maps of DEGs in transitional B cells (A), resting naive B cells (B), unswitched memory B cells (C), resting switched memory B cells (D), and activated switched memory B cells (E) between IgG4-RD and HCs. (F) Heat map of DEGs between activated naive B cells in IgG4-RD and resting naive B cells in IgG4-RD. (G) Heat map of DEGs between activated naive B cells in IgG4-RD and resting naive B cells in healthy controls. (H) The expression level

of *USP25* on total naive B cells and memory B cells in IgG4-RD patients and HCs. (I-L) The expression level of *USP25* on total naive B cells and memory B cells in IgG4-RD and HCs was shown by UMAP separately. Statistical significance was based on wilcoxon rank sum test. Relevant p values are given in the graph. *P < 0.05; **P < 0.01; ***P < 0.001. Source data are provided as a Source Data file.

Supplementary Figure 5

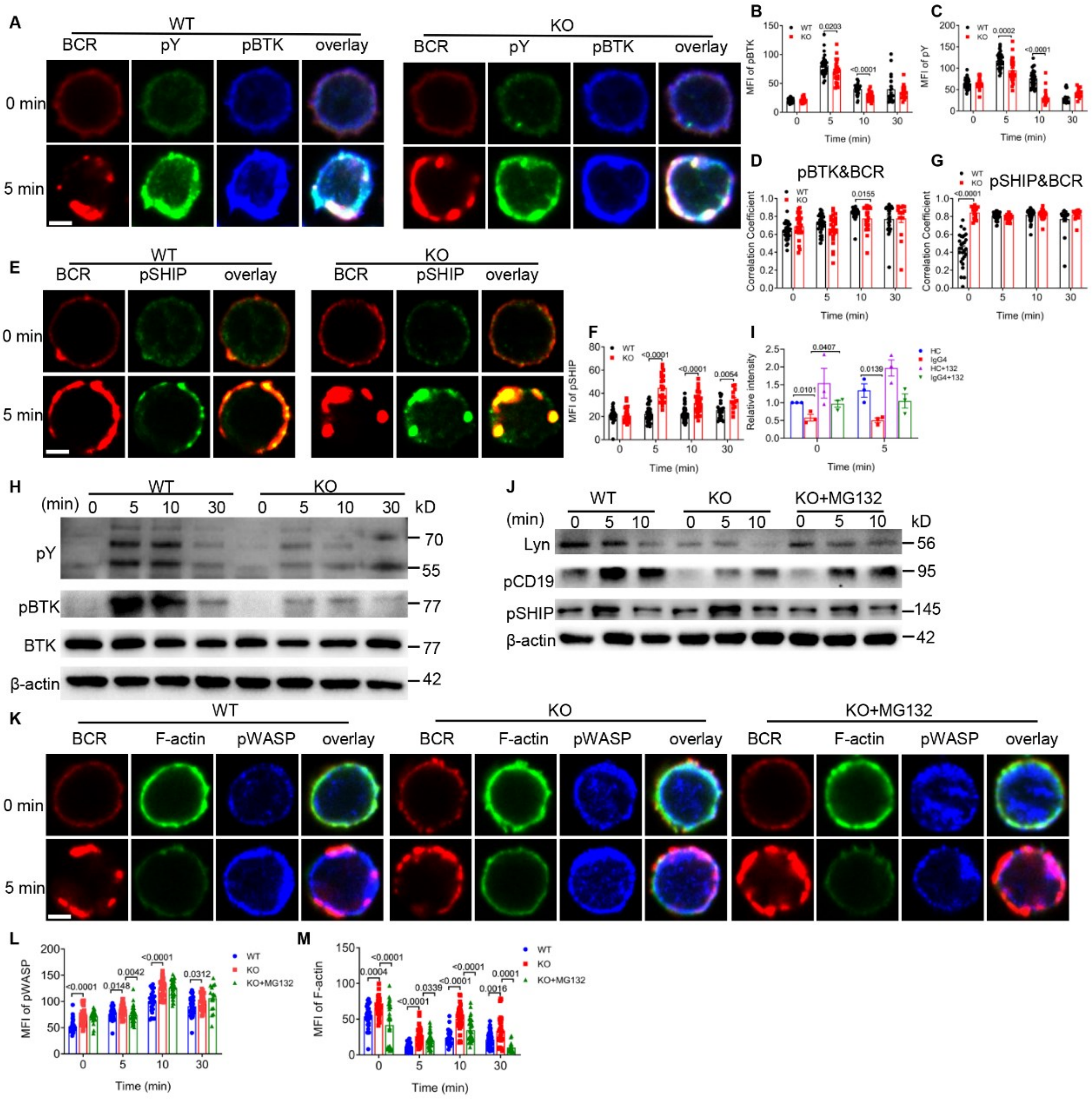

Supplementary Figure 5: USP25 enhances BCR signaling and reduces actin accumulation.

(A-G) Purified B cells from WT and *Usp25* KO mice were stimulated with 10 μg/ml Alexa Fluor 594-F(ab′)$_2$ Ig (M + G), fixed, permeabilized, and stained for pY (A), pBTK (A) and pSHIP (E). A representative image taken using confocal fluorescence microscopy is shown (scale bar = 2.5 μm) and the B cells were analyzed for the MFI of pBTK (B), pY (C), pSHIP (F), and the correlation coefficient of pBTK/BCR (D) and pSHIP/BCR (G) were analyzed. (H) Western blots of pY, pBTK, and BTK in B cells of *Usp25* KO and WT mice stimulated

with 10 μg/ml biotin-F(ab′)$_2$ Ig (M + G) plus 20 μg/ml streptavidin for 5-, 10-, and 30-min. Representative results are shown. (I) The relative protein level of USP25 was measured in PBMC from HCs and IgG4-RD patients by western blots (n=3). (J) Purified B cells from WT and *Usp25* KO mice were treated with 25 μM of MG132 at 37°C for 30 min before incubating with 10 μg/ml of biotin-F(ab′)$_2$-anti-mouse Ig (M + G) plus 20 μg/ml of streptavidin for 5-, and 10-min. Samples were analyzed for the levels of LYN, pCD19, and pSHIP using immunoblotting. Representative results are shown. (K-M) Purified B cells of *Usp25* KO and WT mice were pre-incubated with 25 μM of MG132 at 37°C for 30 min and then incubated with 10 μg/ml AF594-F(ab′)$_2$ Ig (M + G), fixed, permeabilized, and stained for pWASP and F-actin. A representative image taken using confocal fluorescence microscopy is shown (scale bar = 2.5 μm) (K) and the B cells were analyzed for the MFI of pWASP (L) and F-actin (M). All images were representative images from 3 independent experiments. Statistical significance was based on two-tailed unpaired Student’s t-test. Relevant p values are given in the graph. *P < 0.05; **P < 0.01; ***P < 0.001. Source data are provided as a Source Data file.

Supplementary Figure 6

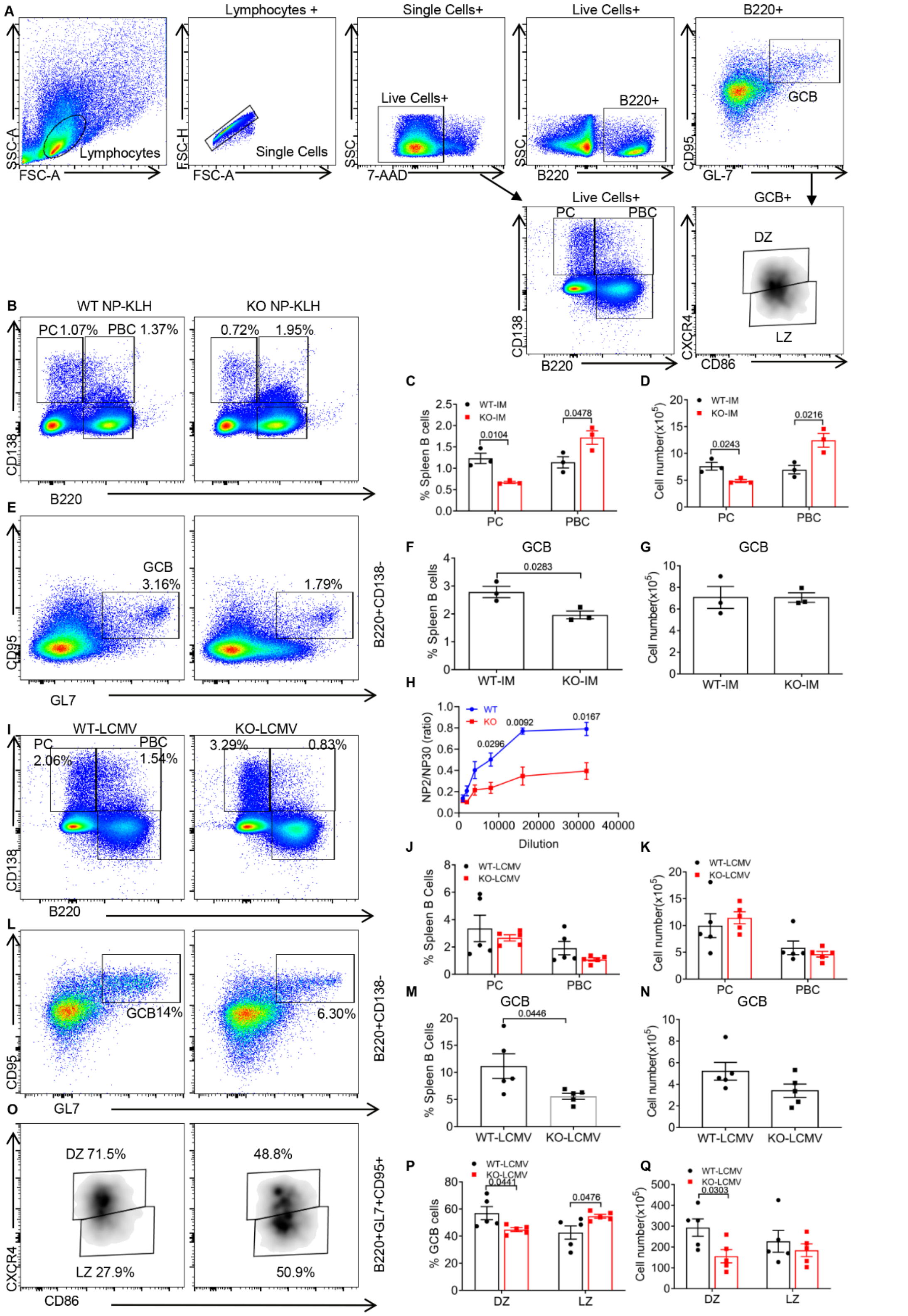

Supplementary Figure 6: USP25 deficiency causes a reduced humoral immune response.

(A) The overall gating strategies for flow cytometry data. (B-G) *Usp25* KO and WT mice were immunized i.p. with 50 μg of 4-NP-KLH precipitated in adjuvant, then the mice were sacrificed after two weeks. Isolated splenic lymphocytes were stained for PC, PBC, and germinal center B cell (GCB), and then analyzed by flow cytometry (C-F), and IM (immunized) (n=3). Shown are representative dot plots (B&E). (H) Serum NP2- and NP30-binding IgG1 were measured by ELISA and the ratio of NP2/NP30 was calculated to reflect the affinity maturation of the antibodies (n = 3). (I-Q) *Usp25* KO and WT mice were given i.p. injection of PBS containing 4×10^5 PFU (in 200 μl) of LCMV-Armstrong 53b, and then the mice were sacrificed a week later. Isolated splenic lymphocytes were stained for PC, PBC, GCB, DZ, and LZ B cells, and then analyzed by flow cytometry (J-K, M-N, P-Q) (n=5). Shown are representative dot plots (I, L, O). Statistical significance was based on two-tailed unpaired Student's t-test. Relevant p values are given in the graph. *P < 0.05; **P < 0.01; ***P < 0.001. Source data are provided as a Source Data file.

Supplementary Figure 7

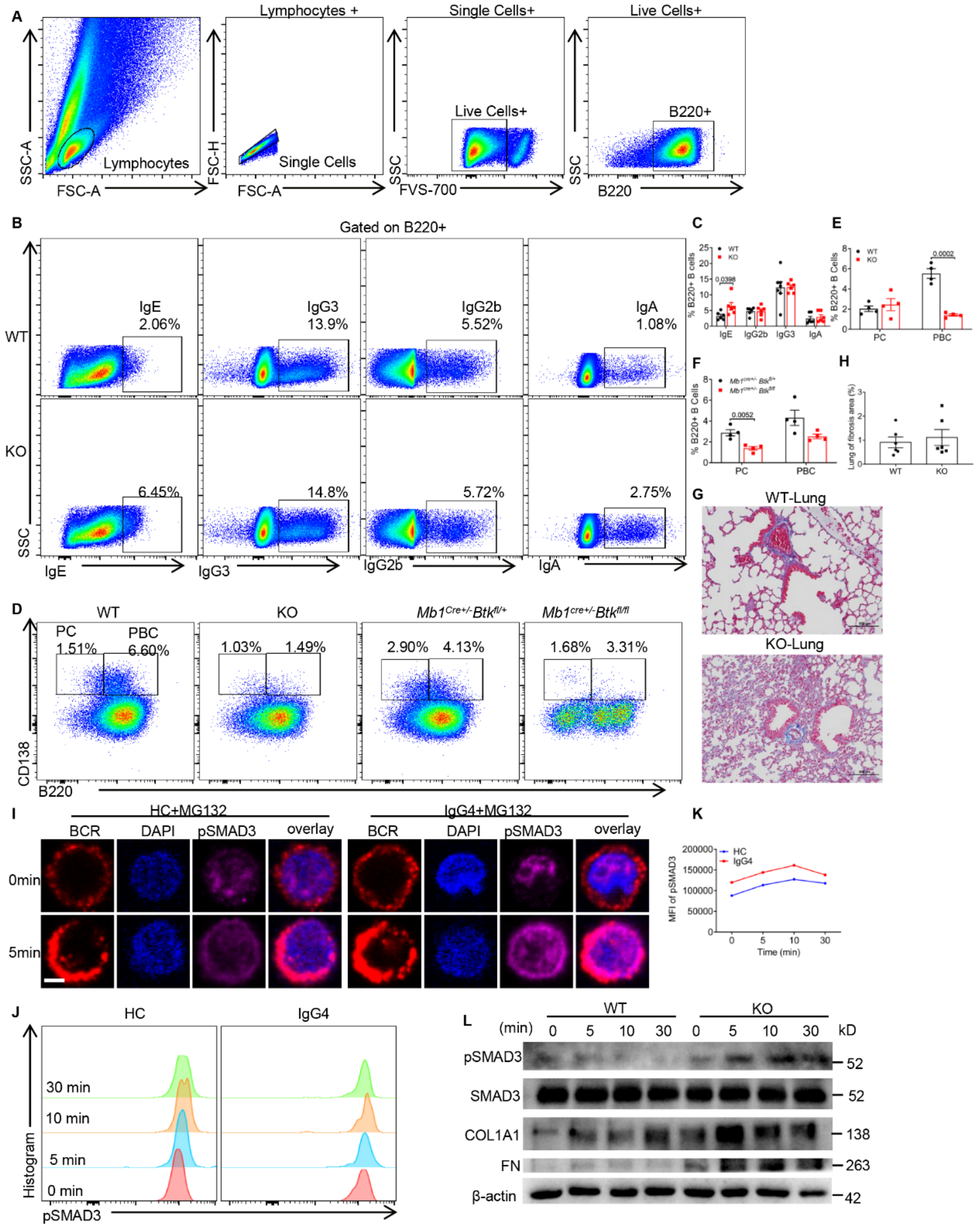

Supplementary Figure 7: USP25 deficiency causes the increase of IgG1 and mediates the upregulation of SMAD3 to induce fibrosis.

(A) The overall gating strategies for flow cytometry data. (B-C) Flow cytometry analysis of IgG3 and IgG2b after stimulating B cells with 10 μg/ml of LPS for 96 h, analysis of IgA after stimulating B cells with 10 μg/ml of LPS plus (0.5 ng/ml)

TGF-β for 120 h, and analysis of IgE after stimulating B cells with 8 ng/ml IL-4 plus 10 μg/ml of CD40 for 120 h (n = 6). (D-F) Flow cytometry analysis of the percentages of PC and PBC in B cells from *Usp25* KO /WT and *Mb1*$^{cre+/-}$*Btk*$^{fl/+}$ / *Mb1*$^{cre+/-}$*Btk*$^{fl/fl}$ mice after stimulating with 10 μg/ml of LPS for 72 h (n=6). (G-H) Masson staining of lung tissues from *Usp25* KO and WT mice and percentage of fibrotic area in the lung of *Usp25* KO and WT mice (n=6, scale bar =100 μm). (I) Purified B cells of IgG4-RD patients and HCs were pre-incubated with 25 μM of MG132 at 37°C for 30 min and then stimulated with 10 μg/ml AF594-F(ab′)$_2$ Ig (M + G), fixed, permeabilized, and stained for pSMAD3 and nuclei using DAPI. A representative image taken using confocal fluorescence microscopy is shown (I) (scale bar = 2.5 μm). (J-K) Cells were analyzed for the MFI of pSMAD3 using flow cytometry and the MFI of pSMAD3 was calculated (n=3). (L) Western blots of pSMAD3, SMAD3, COL1A1 and FN in B cells of *Usp25* KO and WT mice stimulated with sAg for designated times. Representative results are shown. All images were representative images from 3 independent experiments. Statistical significance was based on two-tailed unpaired Student's t-test. Relevant p values are given in the graph. *P < 0.05; **P < 0.01; ***P < 0.001. Source data are provided as a Source Data file.

Supplementary Figure 8

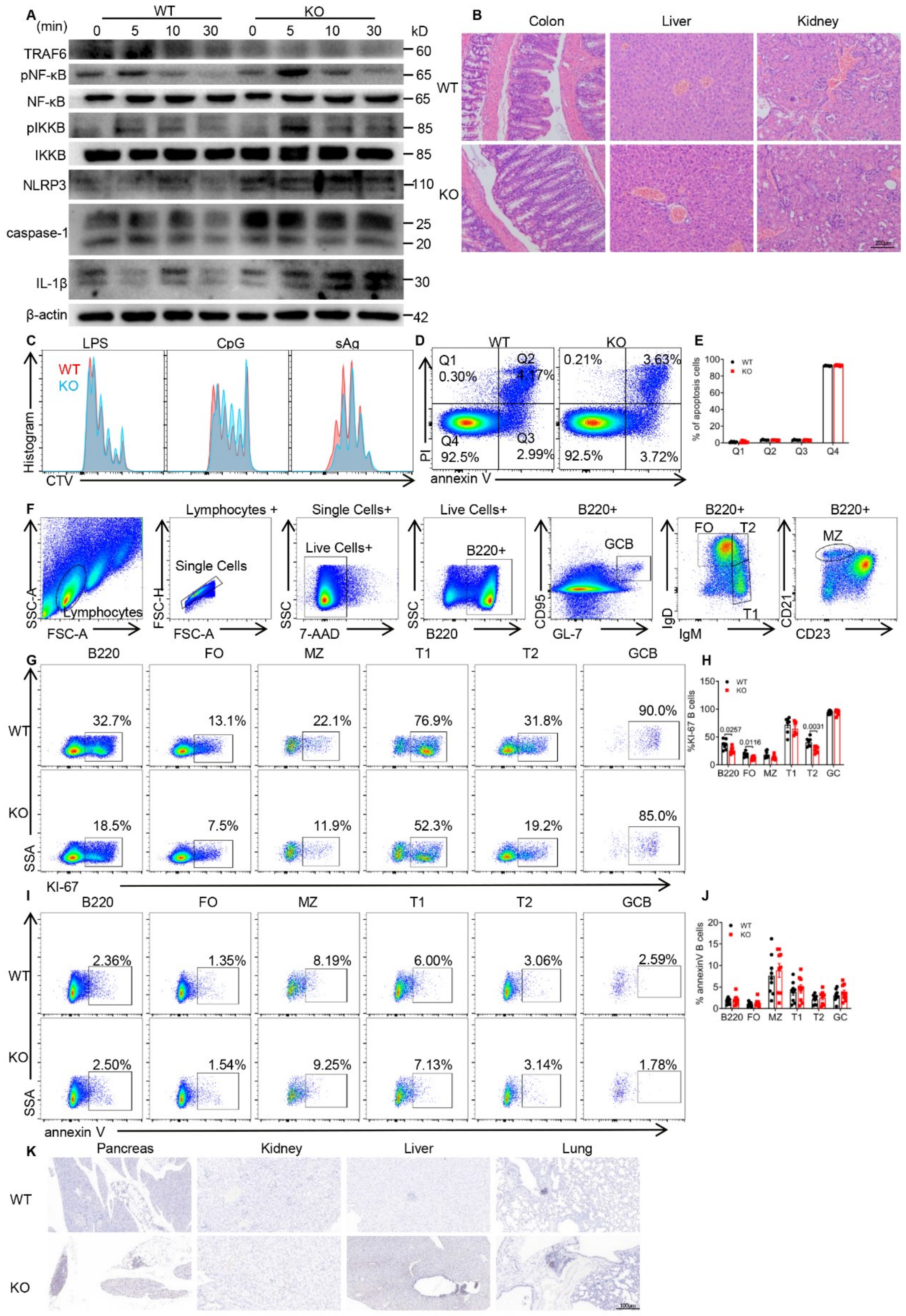

Supplementary Figure 8: USP25 deficiency is mediated via the IL-1β inflammasome axis.

(A) Purified B cells of *Usp25* KO and WT mice were pre-incubated with 10 ug/ml of LPS at 37°C for 24 h and then stimulated with sAg for designated times. Samples were immunoblotted for TRAF6, pNF-κB, pIKKB, NF-κB, IKKB, NLRP3, caspase-1, and IL-1β. Representative results are shown. (B) H&E staining results of colon, liver, and kidney tissues of WT and *Usp25* KO mice after being injected intraperitoneally (i.p.) with 10 mg/kg of LPS for 24 h (scale bar = 200 μm). (C) Purified B cell proliferation in HCs and IgG4-RD patients on day 3 after stimulation with LPS (10 μg/ml), CPG (10 μg/ml), and day 4 after stimulation with sAg (3 μg/ml) (n=3). (D-E) B cells of *Usp25* KO and WT mice were stained with annexin V-FITC/PI and analyzed for the percentage of dead cells (Q1), early apoptotic cells(Q2), late apoptotic cells (Q3) and non-apoptotic cells (Q4) (n=6). (F) The overall gating strategies for flow cytometry data. (G-H) Flow cytometry analysis of the percentages of KI-67 in B cell, transitional 1 (T1), transitional 2 (T2), follicular (FO), marginal zone (MZ) and GCB cells of *Usp25* KO and WT mice. A representative image is shown and the percentages of KI-67 was calculated (n=7). (I-J) Flow cytometry analysis of the percentages of annexin V in B cell, T1, T2, FO, MZ and GC B cells of *Usp25* KO and WT mice. A representative image is shown and the percentages of annexin V was calculated (n=9). (K) CD19 immunohistochemical staining in the pancreas, kidney, liver, and lung of *Usp25* KO and WT mice (scale bar = 100 μm). All images were representative images from 3 independent experiments. Statistical significance was based on two-tailed unpaired Student's t-test. Relevant p values are given in the graph. *P < 0.05; **P < 0.01; ***P < 0.001. Source data are provided as a Source Data file.

Supplementary Figure 9

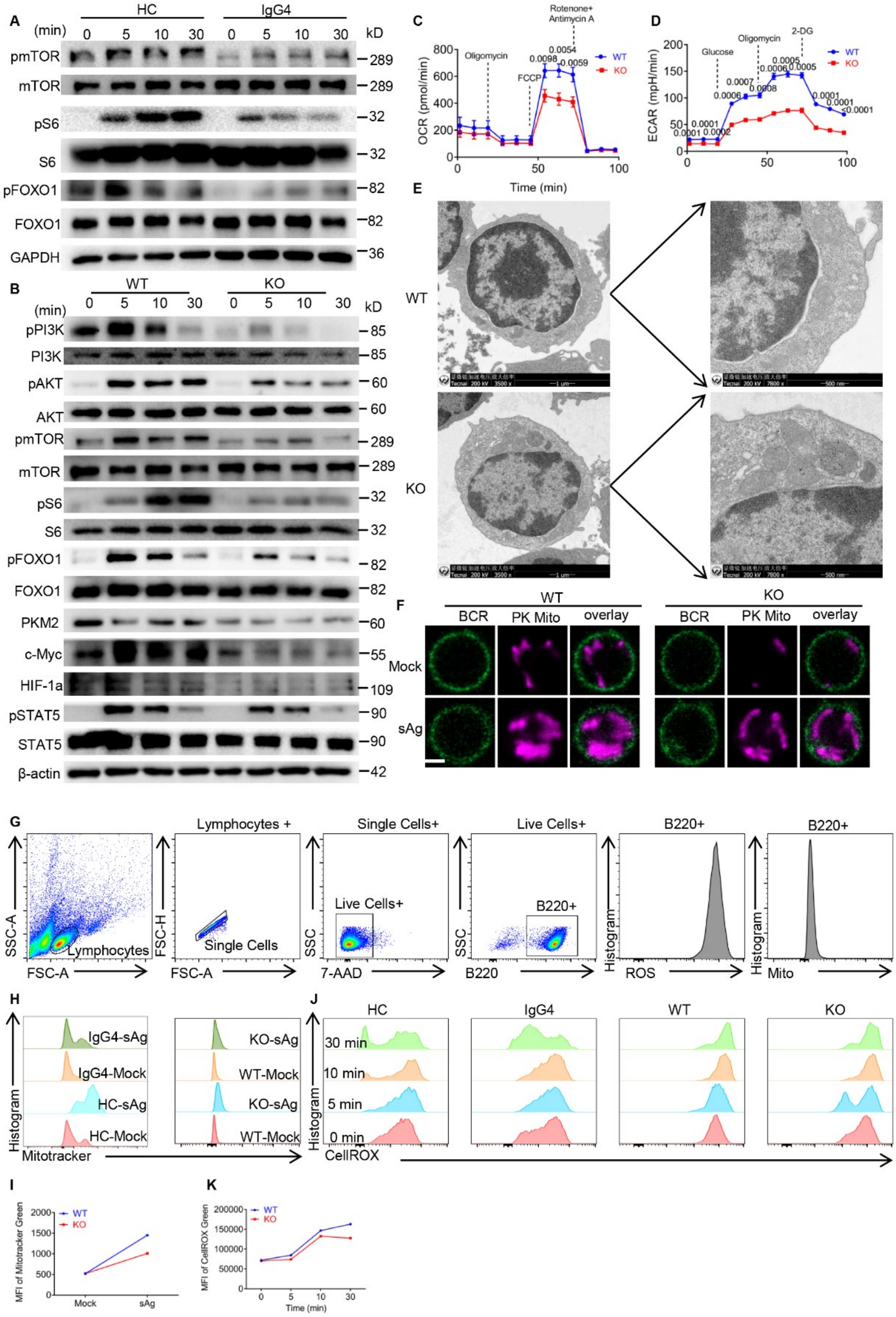

Supplementary Figure 9: USP25 deficiency affects B cell metabolism through the PI3K-AKT pathway.

(A) Western blot of pmTOR, mTOR, pS6, S6, pFOXO1, FOXO1 in PBMCs of IgG4-RD patients and HCs stimulated with sAg for 5-, 10-, and 30-min. Representative results are shown. (B) B cells of *Usp25* KO and WT mice stimulated with sAg for 5-, 10-, and 30- min were analyzed by immunoblotting for pPI3K, pAKT, pmTOR, pS6, pFOXO1, PI3K, AKT, mTOR, S6, FOXO1, PKM2, c-MYC, HIF-1α, pSTAT5 and STAT5. Representative results are shown. (C-D) OCR and ECAR detection in B cells of *Usp25* KO and WT mice. (E) B cells of *Usp25* KO and WT mice were analyzed for disrupted mitochondrial structure using TEM (scale bar = 1 μm-500nm). (F) Purified B cells of *Usp25* KO and WT mice were incubated with 10 μg/ml sAg for 24 h at 37°C, stained for B220 and PK Mito, and analyzed using confocal microscopy for the MFI of PK Mito in B220+ cells (scale bar = 2.5 μm). (G) The overall gating strategies for flow cytometry data. (H-I) PBMCs and purified splenic B cells were stimulated with 10 μg/ml sAg for 24 h at 37°C, stained for CD19 plus mitotracker and B220 plus mitotracker, respectively, and then analyzed for the MFI of mitotracker Green in B cells using flow cytometry. A representative image is shown and the MFI of mitotracker Green was calculated (n=3). (J-K) PBMCs and purified splenic B cells were stimulated with sAg for indicated times, stained for CD19 plus CellROX Green and B220 plus CellROX Green, respectively, and then analyzed for the MFI of CellROX Green in B cells using flow cytometry (n=3). All images were representative images from 3 independent experiments. Statistical significance was based on two-tailed unpaired Student's t-test. Relevant p values are given in the graph. *P < 0.05; **P < 0.01; ***P < 0.001. Source data are provided as a Source Data file.

Supplementary Figure 10

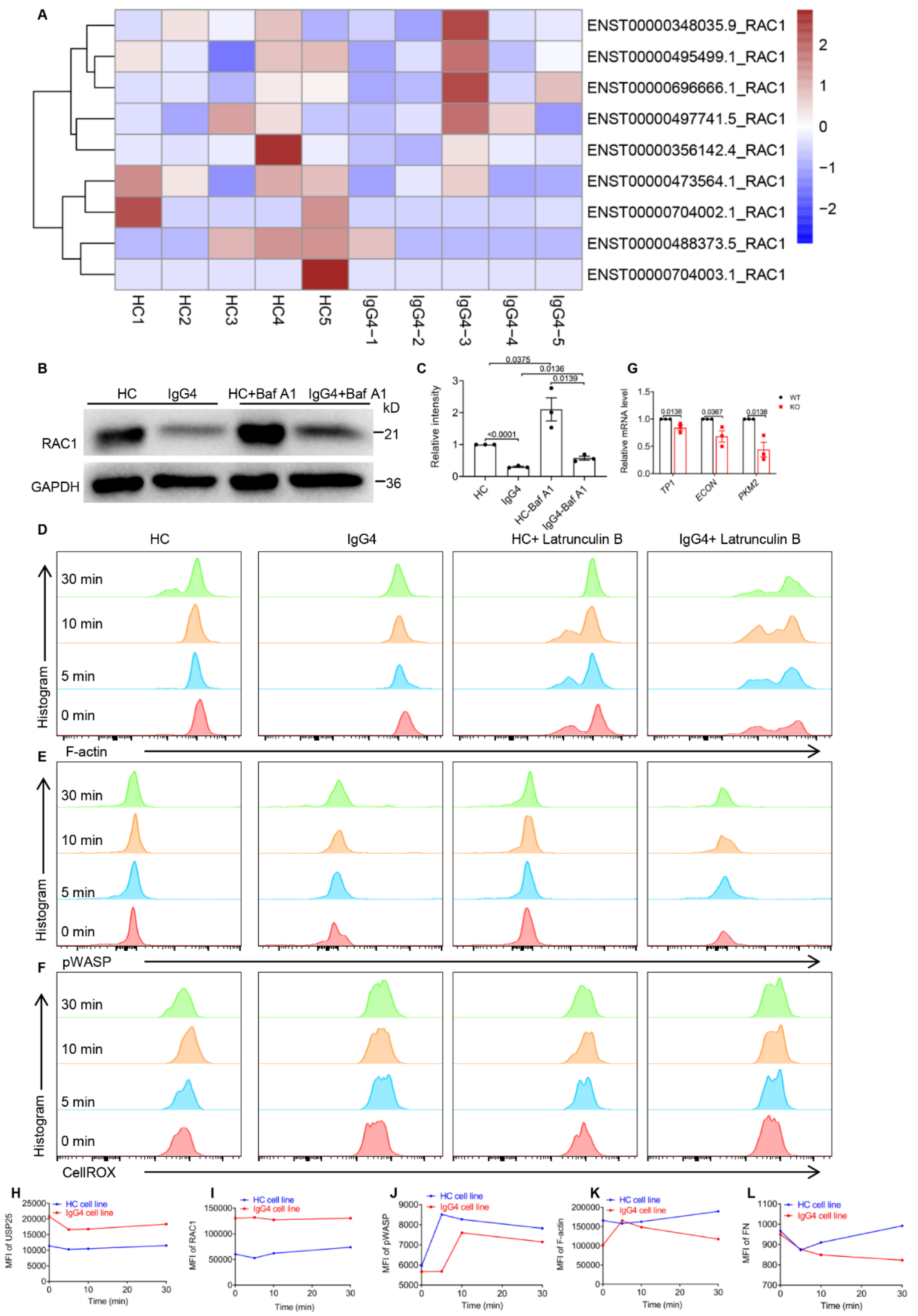

Supplementary Figure 10: RAC1 participates in the regulation of glucose metabolism in B cells.

(A) The expression level of RAC1 transcripts in B cells of IgG4-RD patients and HCs, DETs（|log2 fold change|≥1，p.adj＜0.01）(n=5). (B-C) PBMCs from IgG4-RD patients and HCs were treated with 150 nM bafilomycin A1 at 37°C for 12 h. Samples were analyzed for the levels of RAC1 using immunoblotting (B). Representative results are shown. The relative protein level of RAC1 was measured in PBMC from HCs and IgG4-RD patients(n=3) (C). (D-E) PBMCs of IgG4-RD patients and HCs were pre-incubated with 10 μM of Latrunculin B for 2 h at 37°C and then incubated with CD19, followed by stimulation with 10 μg/ml biotin-F(ab′)$_2$ Ig (M + G) plus 20 μg/ml streptavidin for designated times. PBMCs were stained with F-actin (D) and pWASP (E) after fixation and permeabilization, and analyzed by flow cytometry. A representative image is shown (n=3). (F) PBMCs from HCs and IgG4-RD patients were pre-incubated in the same way as in (Fig.7H) except stained for anti-CD19 and CellROX Green and the MFI of CellROX Green were analyzed. A representative image is shown (n=3). (G) RT-PCR analyzes the mRNA level of *TP1*, *ECON,* and *PKM2* in purified B cells of *Usp25* KO and WT mice (n=3) (H-L) HC cell lines and IgG4-RD cell lines were pre-incubated with anti-CD19 antibody, followed by stimulation with 10 μg/ml sAg plus 20 μg/ml streptavidin for designated times. Then fixed and permeabilized, cells were stained for USP25, F-actin, RAC1, pWASP, and FN and analyzed by flow cytometry. CD19+ B cells were analyzed for the MFI of USP25 (H), RAC1 (I), pWASP (J), F-actin (K), and FN (L) using FlowJo software. All images were representative images from 3 independent experiments. Statistical significance was based on two-tailed unpaired Student's t-test. Relevant p values are given in the graph. *P < 0.05; **P < 0.01; ***P < 0.001. Source data are provided as a Source Data file.

Supplementary Table 2

Basic clinical information of IgG4-RD patients in our study.

ID	Age (years)	Sex	Serum IgG4 (mg/dL)	Responder Index (RI)	Involved organ	Active Disease
1	45	Female	1200	6	Pancreas, biliary tract, lymph nodes	Yes
2	43	Male	280	2	Orbit	Yes
3	56	Male	285	6	Pancreas, submandibular glands, lymph nodes	Yes
4	43	Female	507	8	Submandibular glands, lacrimal glands, salivary glands, lymph nodes	Yes
5	62	Male	1106.8	3	Pancreas, submandibular glands, lymph nodes	Yes
6	53	Male	3100	8	Pancreas, biliary tract, lymph nodes	Yes
7	55	Female	309	2	Submandibular glands,	Yes
8	66	Male	356	2	Retroperitoneum,	Yes
9	52	Female	330	4	Pancreas, submandibular glands, lymph nodes	Yes
10	62	Male	2210	5	Pancreas, biliary tract, lymph nodes	Yes
11	47	Male	1840	3	Lymph nodes	Yes
12	66	Male	1760	4	Lacrimal glands, salivary glands	Yes
13	70	Male	581	2	Retroperitoneum	Yes
14	64	Male	630	5	Pancreas, biliary tract, retroperitoneum	Yes

15	75	Male	1390	10	Pancreas, retroperitoneum, lungs, prostate, lymph nodes	Yes
16	40	Male	1740	7	Pancreas, biliary tract, submandibular glands, lacrimal glands, lymph nodes	Yes
17	54	Male	554	4	Submandibular glands, lacrimal glands,	Yes
18	71	Male	986	6	Pancreas, biliary tract,	Yes
19	87	Male	229	2	Pancreas, biliary tract,	Yes
20	70	Female	394	4	Pancreas, submandibular glands,	Yes
21	44	Male	419	3	Lacrimal glands, lymph nodes	Yes
22	47	Male	280	2	Lymph nodes	Yes
23	70	Male	316	3	Biliary tract, lymph nodes	Yes
24	62	Male	419	5	Pancreas, retroperitoneum, kidney, aorta, lymph nodes	Yes
25	73	Male	893	5	Pancreas, biliary tract, kidney	Yes
26	58	Female	153.7	2	Submandibular glands, lungs	Yes
27	67	Male	1600	4	Pancreas, biliary tract,	Yes
28	65	Male	1311.6	8	Lacrimal glands, salivary glands, prostate, lymph nodes	Yes
29	69	Male	477	3	Pancreas, biliary tract, submandibular glands,	Yes
30	72	Male	305	4	Pancreas, biliary	Yes

					tract, kidney, aorta	
31	43	Male	417	3	Orbit	Yes
32	63	Male	526	4	Submandibular glands, lymph nodes	Yes
33	54	Male	503	3	Submandibular glands, lymph nodes	Yes
34	52	Male	888	5	Pancreas, lacrimal glands, salivary glands,	Yes
35	54	Male	360	2	Pancreas	Yes
36	42	Female	2090	4	Pancreas，Kidney	Yes
37	49	Male	536.9	4	Submandibular glands, lacrimal glands, salivary glands,	Yes
38	51	Male	548	4	Pancreas, submandibular glands, lymph nodes, salivary glands,	Yes
39	50	Male	1060	5	Lymph nodes, kidney, lungs	Yes
40	63	Male	1670	5	Pancreas, submandibular glands, lacrimal glands, lymph nodes	Yes
41	61	Male	1290	4	Pancreas, biliary tract, kidney,	Yes
42	60	Male	1950	4	Submandibular glands, lacrimal glands	Yes
43	53	Male	549	4	Pancreas, biliary tract, submandibular glands, lymph nodes	Yes

Supplementary Table 3: Antibodies used in the study.

Antibody	Supplier	Reference	Dilution
FITC-anti-CD19	Biolegend	Cat#302206, clone: HIB19, RRID: AB_314236	1 μl per $10^{\wedge 6}$ cells
Percp-anti-CD19	Biolegend	Cat#302228, clone: HIB19, RRID: AB_893272	1 μl per $10^{\wedge 6}$ cells
FITC anti-BAFF-R	Biolegend	Cat#316904, clone: 11C1, RRID: AB_528981	1 μl per $10^{\wedge 6}$ cells
FITC anti-CD79α	Biolegend	Cat#333512, clone: HM47, RRID: AB_2565984	1 μl per $10^{\wedge 6}$ cells
PE-anti-CD19	Biolegend	Cat#302208, clone: HIB19, RRID: AB_314238	1 μl per $10^{\wedge 6}$ cells

PE-anti-CD24	Biolegend	Cat#311106, clone: ML5, RRID: AB_314855	1 µl per 10^6 cells
Pacific Blue-anti-CD38	Biolegend	Cat#356628, clone: HB-7, RRID: AB_2629731	1 µl per 10^6 cells
Brilliant Violet 510-anti-IgD	Biolegend	Cat#348220, clone: IA6-2, RRID: AB_2561945	1 µl per 10^6 cells
Alexa Fluor 647 anti-CD27	Biolegend	Cat#302812, clone: O323, RRID: AB_493082	1 µl per 10^6 cells
FITC-anti-CD95	Biolegend	Cat#152606, clone: SA367H8, RRID: AB_2632901	1 µl per 10^6 cells

APC-anti-GL7	Biolegend	Cat#144606, clone: GL7, RRID: AB_2562185	1 µl per 10^6 cells
BV510-anti-B220	Biolegend	Cat#103206, clone: RA3-6B2, RRID: AB_312991	1 µl per 10^6 cells
APC-anti-CD21	Biolegend	Cat#123412, clone: 7E9, RRID: AB_2085160	1 µl per 10^6 cells
PE-anti-CD23	Biolegend	Cat#101608, clone: B3B4, RRID: AB_312833	1 µl per 10^6 cells
FITC-anti-Annexin V	Biolegend	Cat#640906, clone: N/A, RRID: AB_2561292	1 µl per 10^6 cells

Percp-anti-IgD	Biolegend	Cat#405710, clone: 11-26c.2a, RRID: AB_1575113	1 µl per 10^6 cells
BV421-anti-IgM	Biolegend	Cat#406518, clone: RMM-1, RRID: AB_2561444	1 µl per 10^6 cells
PE-anti-NP	Biosearch	Cat#N-5070-1, clone: N/A, RRID: N/A	1 µl per 10^6 cells
BV510-anti-CD138	Biolegend	Cat#142521, clone: 281-2, RRID: AB_2562727	1 µl per 10^6 cells
PE-Cy7-anti-Ki67	eBioscience	Cat#25-5698-82, clone: N/A, RRID: N/A	1 µl per 10^6 cells
Percp-anti-B220	Biolegend	Cat#103234, clone: 103234, RRID: AB_893353	1 µl per 10^6 cells

Anti-IgG1	BD Pharmingen	Cat#757435, clone: A85-1, RRID: N/A	1 µl per 10^6 cells
Anti-IgE	BD Pharmingen	Cat# 757701, clone: R35-72, RRID: N/A	1 µl per 10^6 cells
Anti-IgG2b	BD Pharmingen	Cat#553395, clone: R12-3, RRID: AB_394833	1 µl per 10^6 cells
Anti-IgG3	BD Pharmingen	Cat#553403, clone: R40-82, RRID: AB_394840	1 µl per 10^6 cells
Anti-IgA	BD Pharmingen	Cat#559354, clone: C10-3, RRID: AB_397235	1 µl per 10^6 cells
7-AAD	BD Pharmingen™	Cat# 559925, clone: N/A, RRID: N/A	1:20

Fixable Viability Stain 700	BD Pharmingen™	Cat# 564997, clone: N/A, RRID: N/A	1:20
Anti-pWASP antibody	Bethyl	Cat#A300-205A, clone: N/A, RRID: AB_263404	1:200
Anti-USP25 antibody	Santacruz	Cat#sc-398414, clone: N/A, RRID: N/A	1:200
Anti-RAC1 antibody	Wuhan Fine Test Biotech	Cat#FNab07065, clone: 5D9, RRID: N/A	1:200
Anti-pAKT antibody	Cell Signaling Technology	Cat#9272S, clone: N/A, RRID: AB_329827	1:200
Anti-Fibronectin antibody	ABclonal	Cat#A12977, clone: ARC2692, RRID: AB_2759824	1:200

Anti-pSMAD3 antibody	ABclonal	Cat#AP0548, clone: N/A, RRID: AB_2771541	1:200
Anti-LAMP1 antibody	Santacruz	Cat#sc-20011, clone: H4A3, RRID: AB_626853	1:200
Anti-EEA1 antibody	Cell Signaling Technology	Cat#48453S, clone: E9Q6G, RRID: AB_2920538	1:200
Anti-Aldolase A antibody	ABclonal	Cat#A1142, clone: N/A, RRID: AB_298024	1:200
Anti-pY antibody	merck-millipore	Cat#05-321, clone: 4G10, RRID: AB_309678	1:200

Anti-pBTK antibody	Abcam	Cat#ab52192, clone: N/A, RRID: AB_873715	1:200
Anti-pCD19 antibody	Cell Signaling Technology	Cat#3571S, clone: N/A, RRID: AB_2072836	1:200
Anti-pSHIP antibody	Cell Signaling Technology	Cat#3941S, clone: N/A, RRID: AB_2296062	1:200
Anti-SHIP antibody	Cell Signaling Technology	Cat#2728S, clone: N/A, RRID: AB_2126244	1:200
Anti-BTK antibody	Cell Signaling Technology	Cat#8547S, clone: N/A, RRID: AB_10950506	1:200

Anti-LYN antibody	Santacruz	Cat#sc-7274, clone: H-6, RRID: AB_627897	1:200
Anti-pPI3K antibody	Cell Signaling Technology	Cat#4228S, clone: N/A, RRID: AB_659940	1:200
Anti-PI3K antibody	Cell Signaling Technology	Cat#4292S, clone: N/A, RRID: AB_329869	1:200
Anti-AKT antibody	Cell Signaling Technology	Cat#9272S, clone: N/A, RRID: AB_329827	1:200
Anti-pFOXO1 antibody	Cell Signaling Technology	Cat#9461S, clone: N/A, RRID: AB_329831	1:200

Anti-FOXO1 antibody	Cell Signaling Technology	Cat#2880S, clone: N/A, RRID: AB_2106495	1:200
Anti-pS6 antibody	Cell Signaling Technology	Cat#4856S, clone: N/A, RRID: AB_2181037	1:200
Anti-S6 antibody	Cell Signaling Technology	Cat#2217S, clone: 5G10, RRID: AB_331355	1:200
Anti-pmTOR antibody	Cell Signaling Technology	Cat#5536S, clone: D9C2, RRID: AB_10691552	1:200
Anti-mTOR antibody	Cell Signaling Technology	Cat#2983S, clone: 7C10, RRID: AB_2105622	1:200

Anti-pBLNK antibody	Cell Signaling Technology	Cat#62144S, clone: N/A, RRID: N/A	1:200
Anti-BLNK antibody	Cell Signaling Technology	Cat#36438T, clone: D3P2H, RRID: AB_2799101	1:200
Anti-pSYK antibody	Cell Signaling Technology	Cat#2710S, clone: C87C1, RRID: AB_2197222	1:200
Anti-SYK antibody	Cell Signaling Technology	Cat#13198S, clone: D3Z1E, RRID: AB_2687924	1:200
Anti-CD19 antibody	Cell Signaling Technology	Cat#90176S, clone: D4V4B, RRID: AB_2800152	1:200

Anti-SMAD3 antibody	ABclonal	Cat#A19115, clone: ARC53861, RRID: AB_2862608	1:200
Anti-COL1A1 antibody	ABclonal	Cat#A1352, clone: N/A, RRID: AB_2760381	1:200
Anti-NLRP3 antibody	ABclonal	Cat#A5652, clone: N/A, RRID: AB_2766412	1:200
Anti-Caspase-1 antibody	ABclonal	Cat#A0964, clone: N/A, RRID: AB_2757485	1:200
Anti-IL-1β antibody	ABclonal	Cat#A11369, clone: N/A, RRID: AB_2758528	1:200

Anti-pIKKB antibody	Cell Signaling Technology	Cat#2697S, clone: 16A6, RRID: AB_2079382	1:200
Anti-IKKB antibody	Cell Signaling Technology	Cat#8943S, clone: N/A, RRID: AB_11024092	1:200
Anti-pNF-κb antibody	Cell Signaling Technology	Cat#3031S, clone: N/A, RRID: AB_330559	1:200
Anti-NF-κB antibody	Cell Signaling Technology	Cat#4764S, clone: C22B4, RRID: AB_823578	1:200
Anti-TRAF6 antibody	ABclonal	Cat#A16991, clone: N/A, RRID: AB_2772697	1:200

Anti-HIF1-α antibody	Active Motif	Cat#39665, clone: N/A, RRID: AB_2614934	1:200
Anti-c-MYC antibody	Cell Signaling Technology	Cat#13987S, clone: D3N8F, RRID: AB_2631168	1:200
Anti-pSTAT5 antibody	Cell Signaling Technology	Cat#4322, clone: N/A, RRID: AB_10544692	1:200
Anti-STAT5 antibody	Cell Signaling Technology	Cat#25656, clone: D3N2B, RRID: AB_2798908	1:200
Anti-PKM2 antibody	Cell Signaling Technology	Cat#4053T, clone: D78A4, RRID: AB_1904096	1:200

Anti-Ubiquitin antibody	SantaCruz	Cat#sc-8017, clone: P4D1, RRID: AB_628423	1μg per IP
Anti-IgG Light Chain antibody	Abbkine	Cat#A25012, clone: N/A, RRID: AB_2737290	1μg per IP
Anti-IgG antibody	SantaCruz	Cat#sc-2025, clone: N/A, RRID: AB_737182	1μg per IP
Alexa Fluor 488 goat anti-rabbit IgG	Thermo Fisher	Cat#A-11008, clone: N/A, RRID: AB_143165	1:400
Alexa Fluor 405 goat anti-rabbit IgG	Thermo Fisher	Cat#A-31556, clone: N/A, RRID: AB_221605	1:400

Alexa Fluor 647 goat anti-rabbit IgG	Thermo Fisher	Cat#A-21245, clone: N/A, RRID: AB_2535813	1:400
Alexa Fluor 488 goat anti-mouse IgG	Jackson ImmunoResearch	Cat#715-165-151, clone: N/A, RRID: AB_2315777	1:400
Alexa Fluor 594-F(ab′)2 goat anti-mouse IgM+IgG (H+L)	Jackson ImmunoResearch	Cat#115-586-068, clone: N/A, RRID: AB_2338895	1:100
Alexa Fluor 594-F(ab′)2 anti-human Ig (M + G)	Jackson ImmunoResearch	Cat#109-586-127, clone: N/A, RRID: AB_2337876	1:100
Biotin-F(ab′)2 anti-human Ig (M + G)	Jackson ImmunoResearch	Cat#109-066-127, clone: N/A, RRID: AB_2337641	1:100

Biotin-conjugated F(ab′)2 anti-mouse Ig (M + G)	Jackson ImmunoResearch	Cat#115-066-068, clone: N/A, RRID: AB_2338581	1:100
IgM-specific secondary antibody	Bethyl	Cat#A90-101P, clone: N/A, RRID: AB_67189	1:5000
IgG1-specific secondary antibody	Bethyl	Cat#A90-105P, clone: N/A, RRID: AB_67150	1:5000
Anti-β-actin antibody	Proteintech	Cat#60008-1-IG-10, clone: 7D2C10, RRID: AB_2289225	1:1000
Anti-GAPDH antibody	Proteintech	Cat#60004-1-IG, clone: 1E6D9, RRID: AB_2107436	1:1000

Supplementary Table 4: Reagents and critical commercial assays used in the study.

Reagent	Supplier	Reference
Fc blocker	BioLegend	Cat#101319
Anti-Thy1.2 mAb	Biolegend	Cat#105310
Ficoll	GE Healthcare	Cat#17-1440-02
CD19 magnetic beads	Miltenyi Biotec	Cat#130-050-301
Guinea pig complement	Rockland	Cat#C300-0500
GolgiStop	BD Biosciences	Cat#554724
Lonomycin	CST	Cat#9995S
AF488-phalloidin	Thermo Fisher	Cat#R37110
LPS	Sigma	Cat#L2880
IL4	Peprotech	Cat#214-14
Celltrace Violet	Thermo Fisher	Cat#C34557
Fetal bovine serum	Pan biotech	Cat#P30-3300
CellROX Green	Invitrogen	Cat#C10444
MitoTracker Green FM	Invitrogen	Cat#M7514
Paraformaldehyde	Thermo Fisher	Cat#28908
Saponin	Sigma	Cat#S4521-10G
AF647-probes for mitochondria	Invitrogen	Cat#M7512

PK Mito Red	Genvivotech	Cat#PKMDR-1
AF405-ER-Tracker dyes	Invitrogen	Cat#E12353
RIPA buffer	Beyotime	Cat#P0013B
Protease inhibitor cocktail	Servicebio	Cat#G2006
MG132	Selleck	Cat#s2619
Bafilomycin A1	TargetMol	Cat#s T6740
Protein G Sepharose	GE Healthcare	Cat#10253638
SDS loading buffer	Biosharp	Cat#BL517A
Digitonin	Solarbio	Cat#ID0410
Poly-d-lysine solution	Beyotime	Cat#C0312
2.5% Glutaraldehyde buffer	Solarbio	Cat#P1126
Oligomyciine	Absin	Cat#abs42024304
Fluoro-carbonylcyanide phenylhydrazone	Sigma	Cat#C2920
Rotenone	Sigma	Cat#R8875
Antimycine	Absin	Cat#abs4201340
Glucose	Sigma	Cat#G8769
NP-KLH	Biosearch	Cat#N-5060-25
Adjuvant (MPL+TDM)	Sigma	Cat#S6322

NP-bovine serum albumin (NP-30)	Biosearch	Cat#N-5050H-100
NP-BSA (Bovine Serum Albumin), Ratio 1-4 (NP-2)	Biosearch	Cat#N-5050XL
TMB Substrate	BD OptEIA	Cat#N-555214
Trizol	Invitrogen	Cat#15596026
Attune performance tracking beads	Invitrogen	Cat#2029773
AxyPrep Multisource RNA Kit	Axygen	Cat#AP-MN-MS-RNA-50
Fixation/Permeabilization Kit	eBioscience	Cat#00-5123, Cat#00-5223
Mouse IgG1 ELISA Kit	NeoBioscience	Cat#EMC130.96
Mouse IL-1β ELISA kit	QuantiCyto®	Cat#EMC001b.96
Mouse IL-13 ELISA kit	QuantiCyto®	Cat# EMC124.96
Glutamic assay Kit	Solarbio	Cat#BC1585
Cysteine assay Kit	Solarbio	Cat#BC0185
PrimeScript RT Reagent Kit	Takara	Cat#RR047A
SYBR Premix Ex TaqTM	Takara	Cat#RR420A
QIAamp DNA Micro Kit	QIAamp	Cat#56304

The HiScript® III 1st Strand cDNA Synthesis Kit	Vazyme	Cat#R211-02
HiPure Universal RNA Mini Kit	Guangzhou Magen Biotechnology	Cat#R4130-02
RNA Nano 6000 Assay Kit	Agilent	Cat#5067-1512
DAB Kit	Servicebio	Cat#G1212
FlowJo	FlowJo	https://www.flowjo.com
NIS Elements	Nikon Instruments	https://www.microscope.healthcare.nikon.com
Chemi Doc™XRS + imaging systems	Bio-Rad	https://www.bio-rad.com
Image Lab™ software	Bio-Rad	https://www.bio-rad.com
Seahorse XF24 analyzer	Agilent	https://www.agilent.com
StepOne Real-Time PCR system	Applied Biosystems	https://qa1.thermofisher.cn/order/catalog/product/cn/en/4376357
GenoLab M sequencing platform	GeneMind Biosciences	http://www.genemind.com
Cutadapt	Cutadapt	https://cutadapt.readthedocs.io/en/stable/

HISAT2	Johns Hopkins University Central for Computational Biology	https://daehwankimlab.github.io/hisat2/
StringTie	Johns Hopkins University Central for Computational Biology	http://ccb.jhu.edu/software/stringtie/index.shtml?t=manual
R	The R Project for Statistical Computing	https://www.r-project.org
Progenesis QI	Waters	https://www.nonlinear.com
metaX	BGI	http://metax.genomics.cn/
GraphPad Prism 7	GraphPad	https://www.graphpad.com/

Supplementary Table 5: Primer sequences used in the study.

Primer	Forward	Reverse
GAPDH (Mouse)	GGTGAAGGTCGGTGTGAACG	CTCGCTCCTGGAAGATGGTG
TP1 (Mouse)	CCAGGAAGTTCTTCGTTGGGG	CAAAGTCGATGTAAGCGGTGG
ECON (Mouse)	TGCGTCCACTGGCATCTAC	CAGAGCAGGCGCAATAGTTTTA
PKM2 (Mouse)	GCCGCCTGGACATTGACTC	CCATGAGAGAAATTCAGCCGAG
RAC1 (Mouse)	GCCGATTGCCGACGTGTT	GTCTTGAGTCCTCGCTGTGT
GLTγ1 (Mouse)	CAGCCTGGTGTCAACTAG	CTGTACATATGCAAGGCT
GAPDH (Human)	ACCCAGAAGACTGTGGATGG	TTCTAGACGGCAGGTCAGGT
BLNK (Human)	AGAGGCTTACCATGCTGCTC	AGGGCTCTCTGAAGCGTAGT
RAC1 (Human)	GTGGGAGACGGAGCTGTAG	AGAACACATCTGTTTGCGGA
TP1 (Human)	CAAACAGGCTGAGCGATTTGGG	GGGAGCACAAACCACCTCTCC

ECON (Human)	GAGACCCAGTGGCTAGAAGT	ATGGGCTGTGGGTTCTAAGG
PKM2 (Human)	ATGCAGCACCTGATAGCTCG	CCATGAGGTCTGTGGAGTGAC
USP25 (Human)	CGGTCCCAAACGATTCCC	CTCCCTGTTCTGTTGTGCT
