# Supplementary material for: Expression of USP25 associates with fibrosis, inflammation and metabolism changes in IgG4-related disease
Source: Nat Commun. 2024 Mar 23;15:2627. doi: 10.1038/s41467-024-45977-7 (PMC10960850; doi:10.1038/s41467-024-45977-7)
Supplement: Supplementary file 3 — Description of Additional Supplementary Files [file 41467_2024_45977_MOESM3_ESM.pdf]

Description of Additional Supplementary Files

File Name: Supplementary Data 1
Description: The RNA-seq data of HCs and IgG4-RD patients B cells
