# Supplementary material for: Expression of USP25 associates with fibrosis, inflammation and metabolism changes in IgG4-related disease
Source: Nat Commun. 2024 Mar 23;15:2627. doi: 10.1038/s41467-024-45977-7 (PMC10960850; doi:10.1038/s41467-024-45977-7)
Supplement: Supplementary file 5 — Reporting Summary [file 41467_2024_45977_MOESM5_ESM.pdf]

nature portfolio

Corresponding author(s): Chaohong Liu

Last updated by author(s): Feb 4, 2024

Reporting Summary

Nature Portfolio wishes to improve the reproducibility of the work that we publish. This form provides structure for consistency and transparency in reporting. For further information on Nature Portfolio policies, see our Editorial Policies and the Editorial Policy Checklist.

Statistics

For all statistical analyses, confirm that the following items are present in the figure legend, table legend, main text, or Methods section.

n/a	Confirmed	
☐	☒	The exact sample size (*n*) for each experimental group/condition, given as a discrete number and unit of measurement
☐	☒	A statement on whether measurements were taken from distinct samples or whether the same sample was measured repeatedly
☐	☒	The statistical test(s) used AND whether they are one- or two-sided<br>*Only common tests should be described solely by name; describe more complex techniques in the Methods section.*
☐	☒	A description of all covariates tested
☒	☐	A description of any assumptions or corrections, such as tests of normality and adjustment for multiple comparisons
☐	☒	A full description of the statistical parameters including central tendency (e.g. means) or other basic estimates (e.g. regression coefficient) AND variation (e.g. standard deviation) or associated estimates of uncertainty (e.g. confidence intervals)
☐	☒	For null hypothesis testing, the test statistic (e.g. *F*, *t*, *r*) with confidence intervals, effect sizes, degrees of freedom and *P* value noted<br>*Give P values as exact values whenever suitable.*
☒	☐	For Bayesian analysis, information on the choice of priors and Markov chain Monte Carlo settings
☒	☐	For hierarchical and complex designs, identification of the appropriate level for tests and full reporting of outcomes
☒	☐	Estimates of effect sizes (e.g. Cohen's *d*, Pearson's *r*), indicating how they were calculated

Our web collection on statistics for biologists contains articles on many of the points above.

Software and code

Policy information about availability of computer code

Data collection	Flow cytometry: Attune™ NxT, AFC2 (Thermo Fisher), BD FACS Aria SORP (BD, FACS Aria SORP)<br>Western blot: ChemiDoc™XRS+imaging systems (Bio-Rad, 1708265)<br>Confocal and total internal reflection fluorescence microscope: Nikon Eclipse Ti-PFS (NCS6112C, Tokyo, Japan)<br>Scanning electron microscopy: SU8010 (HITACHI, Japan)<br>Transmission electron microscopy: H-7000FA (HITACHI, Japan)<br>Seahorse XFe24 Analyzer: Agilent Seahorse XFe24 (USA)<br>ELISA: INFINITE 200 PRO(TECAN)<br>qTOWER³ Real-Time PCR Thermal Cycler: Jena, 844-00553-2
Data analysis	GraphPad Prism (8.0.2), R package clusterProfiler (4.8.1), Image Lab (3.0 39529), Image J(1.8.0), FlowJo (V10), NIS-elements (AR 5.01), qPCRsoft (4.1), Seahorse Wave (2.6.1), Progenesis QI (version 2.2), CellRanger (v7.1.0), Seurat (v4.3.0) software were used to analyze data.

For manuscripts utilizing custom algorithms or software that are central to the research but not yet described in published literature, software must be made available to editors and reviewers. We strongly encourage code deposition in a community repository (e.g. GitHub). See the Nature Portfolio guidelines for submitting code & software for further information.

Data

Policy information about availability of data

All manuscripts must include a data availability statement. This statement should provide the following information, where applicable:

- Accession codes, unique identifiers, or web links for publicly available datasets
- A description of any restrictions on data availability
- For clinical datasets or third party data, please ensure that the statement adheres to our policy

The mouse RNA-Seq data generated in this study have been deposited in Genome Sequence Archive (GSA) database under accession code CRA014878. The human RNA-Seq data generated in this study have been deposited in GSA database under accession code HRA006661. The Single-Cell RNA-sequencing data generated in this study have been deposited in GSA database under accession code HRA001555. Uncropped western blots for data in main and Supplementary Figs. are provided in the Source data file. Source data are provided with this paper.

Research involving human participants, their data, or biological material

Policy information about studies with human participants or human data. See also policy information about sex, gender (identity/presentation), and sexual orientation and race, ethnicity and racism.

Reporting on sex and gender	No gender specific analyses were performed in this study. The trial recruited female and male patients without any prejudice. Most patients in the trial were male (n=36/43; 83.72%) since IgG4-RD is a male predominated disease.
Reporting on race, ethnicity, or other socially relevant groupings	There was no bias of recruitment in terms of race, ethnicity, or other socially relevant.
Population characteristics	IgG4-RD patients and healthy control study participants were matched for age (range 42–87 years old), sex and ethnicity in the discovery. IgG4-RD patients were eligible if they had dense IgG4+ lymphoplasmocytes infiltrates, storiform pattern of fibrosis and obliterans phlebitis at study entry.
Recruitment	IgG4-RD patients and healthy controls were recruited sequentially from department of rheumatology and Immunology and other pre-operative clinics at Tongji Hospital, Tongji Medical College, Huazhong University of Science and Technology. Control study participants were chosen to match to each IgG4-RD patients participant (age within 3-yrs, sex). Study candidates were identified by treating physicians at Department of Rheumatology and Immunology, Tongji Hospital. To avoid any self-selection bias or other biases, all participants included in this study signed an informed consent waiver to contribute de-identifie.
Ethics oversight	The study was approved by the Huazhong University of Science, and Technology Institutional Review Board Approval (reference TJ-IRB20220434)

Note that full information on the approval of the study protocol must also be provided in the manuscript.

Field-specific reporting

Please select the one below that is the best fit for your research. If you are not sure, read the appropriate sections before making your selection.

☒ Life sciences ☐ Behavioural & social sciences ☐ Ecological, evolutionary & environmental sciences

For a reference copy of the document with all sections, see nature.com/documents/nr-reporting-summary-flat.pdf

Life sciences study design

All studies must disclose on these points even when the disclosure is negative.

Sample size	No sample size calculation was performed given the rarity of IgG4-related disease. Sample sizes were selected based on the previous experience and published literature to detect meaningful biological differences (https://www.sciencedirect.com/science/article/pii/S0896841122001524?via%3Dihub#appsec1). All identified patient samples meeting diagnostic criteria were included in this study and all experiments were performed in biological triplicate to ensure reproducibility.
Data exclusions	No data was excluded from the analysis.
Replication	All attempts at replication were successful. Data were representative of 3 independent experiments.
Randomization	All experiments were conducted using randomly selected samples of IgG4-RD patients and all samples were randomly grouped before the experiment . For mouse experiments, animals were randomized. For cell line experiments, randomization was not relevant and B-cell lines were specifically generated to either express or do not express USP25.

Blinding

Investigators were blinded to group allocation during data collection, image quantification and data analysis.

Reporting for specific materials, systems and methods

We require information from authors about some types of materials, experimental systems and methods used in many studies. Here, indicate whether each material, system or method listed is relevant to your study. If you are not sure if a list item applies to your research, read the appropriate section before selecting a response.

Materials & experimental systems

n/a	Involved in the study	
☐	☒	Antibodies
☐	☒	Eukaryotic cell lines
☒	☐	Palaeontology and archaeology
☐	☒	Animals and other organisms
☒	☐	Clinical data
☒	☐	Dual use research of concern
☒	☐	Plants

Methods

n/a	Involved in the study	
☒	☐	ChIP-seq
☐	☒	Flow cytometry
☒	☐	MRI-based neuroimaging

Antibodies

Antibodies used

Antibody / Source/Catalog #/Clone / RRID/Dilution
FITC-anti-CD19 Biolegend Cat#302206, clone: HIB19, RRID: AB_314236 1µl per 10^6 cells
Percp-anti-CD19 Biolegend Cat#302228, clone: HIB19, RRID: AB_893272 1µl per 10^6 cells
FITC anti-BAFF-R Biolegend Cat#316904, clone: 11C1, RRID: AB_528981 1µl per 10^6 cells
FITC anti-CD79α Biolegend Cat#333512, clone: HM47, RRID: AB_2565984 1µl per 10^6 cells
PE-anti-CD19 Biolegend Cat#302208, clone: HIB19, RRID: AB_314238 1µl per 10^6 cells
PE-anti-CD24 Biolegend Cat#311106, clone: ML5, RRID: AB_314855 1µl per 10^6 cells
Pacific Blue-anti-CD38 Biolegend Cat#356628, clone: HB-7, RRID: AB_2629731 1µl per 10^6 cells
Brilliant Violet 510-anti-IgD Biolegend Cat#348220, clone: IA6-2, RRID: AB_2561945 1µl per 10^6 cells
Alexa Fluor 647 anti-CD27 Biolegend Cat#302812, clone: O323, RRID: AB_493082 1µl per 10^6 cells
FITC-anti-CD95 Biolegend Cat#152606, clone: SA367H8, RRID: AB_2632901 1µl per 10^6 cells
APC-anti-GL7 Biolegend Cat#144606, clone: GL7, RRID: AB_2562185 1µl per 10^6 cells
BV510-anti-B220 Biolegend Cat#103206, clone: RA3-6B2, RRID: AB_312991 1µl per 10^6 cells
APC-anti-CD21 Biolegend Cat#123412, clone: 7E9, RRID: AB_2085160 1µl per 10^6 cells
PE-anti-CD23 Biolegend Cat#101608, clone: B3B4, RRID: AB_312833 1µl per 10^6 cells
FITC-anti-Annexin V Biolegend Cat#640906, clone: N/A, RRID: AB_2561292 1µl per 10^6 cells
Percp-anti-IgD Biolegend Cat#405710, clone: 11-26c.2a, RRID: AB_1575113 1µl per 10^6 cells
BV421-anti-IgM Biolegend Cat#406518, clone: RMM-1, RRID: AB_2561444 1µl per 10^6 cells
PE-anti-NP Biosearch Cat#N-5070-1, clone: N/A, RRID: N/A 1µl per 10^6 cells
BV510-anti-CD138 Biolegend Cat#142521, clone: 281-2, RRID: AB_2562727 1µl per 10^6 cells
PE-Cy7-anti-Ki67 eBioscience Cat#25-5698-82, clone: N/A, RRID: N/A 1µl per 10^6 cells
Percp-anti-B220 Biolegend Cat#103234, clone: 103234, RRID: AB_893353 1µl per 10^6 cells
Anti-IgG1 BD Pharmingen Cat#757435, clone: A85-1, RRID: N/A 1µl per 10^6 cells
Anti-IgE BD Pharmingen Cat# 757701, clone: R35-72, RRID: N/A 1µl per 10^6 cells
Anti-IgG2b BD Pharmingen Cat#553395, clone: R12-3, RRID: AB_394833 1µl per 10^6 cells
Anti-IgG3 BD Pharmingen Cat#553403, clone: R40-82, RRID: AB_394840 1µl per 10^6 cells
Anti-IgA BD Pharmingen Cat#559354, clone: C10-3, RRID: AB_397235 1µl per 10^6 cells
7-AAD BD Pharmingen™ Cat# 559925, clone: N/A, RRID: N/A 1µl per 10^6 cells
Fixable Viability Stain 700 BD Pharmingen™ Cat# 564997, clone: N/A, RRID: N/A 1µl per 10^6 cells
Anti-pWASP antibody Bethyl Cat#A300-205A, clone: N/A, RRID: AB_263404 1:200
Anti-USP25 antibody Santacruz Cat#sc-398414, clone: N/A, RRID: N/A 1:200
Anti-RAC1 antibody Wuhan Fine test Biotech Cat#FNab07065, clone: 5D9, RRID: N/A 1:200
Anti-pAKT antibody Cell Signaling Technology Cat#9272S, clone: N/A, RRID: AB_329827 1:200
Anti-Fibronectin antibody ABclonal Cat#A12977, clone: ARC2692, RRID: AB_2759824 1:200
Anti-pSMAD3 antibody ABclonal Cat#AP0548, clone: N/A, RRID: AB_2771541 1:200
Anti-LAMP1 antibody Santacruz Cat#sc-20011, clone: H4A3, RRID: AB_626853 1:200
Anti-EEA1 antibody Cell Signaling Technolog Cat#48453S, clone: E9Q6G, RRID: AB_2920538 1:200
Anti-Aldolase A antibody ABclonal Cat#A1142, clone: N/A, RRID: AB_298024 1:200
Anti-pY antibody merck-millipore Cat#05-321, clone: 4G10, RRID: AB_309678 1:200
Anti-pBTK antibody abcam Cat#ab52192, clone: N/A, RRID: AB_873715 1:200
Anti-pCD19 antibody Cell Signaling Technology Cat#3571S, clone: N/A, RRID: AB_2072836 1:200
Anti-pSHIP antibody Cell Signaling Technology Cat#3941S, clone: N/A, RRID: AB_2296062 1:200
Anti-SHIP antibody Cell Signaling Technology Cat#2728S, clone: N/A, RRID: AB_2126244 1:200
Anti-BTK antibody Cell Signaling Technology Cat#8547S, clone: N/A, RRID: AB_10950506 1:200
Anti-LYN antibody Santacruz Cat#sc-7274, clone: H-6, RRID: AB_627897 1:200
Anti-pPI3K antibody Cell Signaling Technology Cat#4228S, clone: N/A, RRID: AB_659940 1:200
Anti-PI3K antibody Cell Signaling Technology Cat#4292S, clone: N/A, RRID: AB_329869 1:200
Anti-AKT antibody Cell Signaling Technology Cat#9272S, clone: N/A, RRID: AB_329827 1:200

Anti-pFOXO1 antibody Cell Signaling Technology Cat#9461S, clone: N/A, RRID: AB_329831 1:200
Anti-FOXO1 antibody Cell Signaling Technology Cat#2880S, clone: N/A, RRID: AB_2106495 1:200
Anti-pS6 antibody Cell Signaling Technology Cat#4856S, clone: N/A, RRID: AB_2181037 1:200
Anti-S6 antibody Cell Signaling Technology Cat#2217S, clone: 5G10, RRID: AB_331355 1:200
Anti-pmTOR antibody Cell Signaling Technology Cat#5536S, clone: D9C2, RRID: AB_10691552 1:200
Anti-mTOR antibody Cell Signaling Technology Cat#2983S, clone: 7C10, RRID: AB_2105622 1:200
Anti-pBLNK antibody Cell Signaling Technology Cat#62144S, clone: N/A, RRID: N/A 1:200
Anti-BLNK antibody Cell Signaling Technology Cat#36438T, clone: D3P2H, RRID: AB_2799101 1:200
Anti-pSYK antibody Cell Signaling Technology Cat#2710S, clone: C87C1, RRID: AB_2197222 1:200
Anti-SYK antibody Cell Signaling Technology Cat#13198S, clone: D3Z1E, RRID: AB_2687924 1:200
Anti-CD19 antibody Cell Signaling Technology Cat#90176S, clone: D4V4B, RRID: AB_2800152 1:200
Anti-SMAD3 antibody ABclonal Cat#A19115, clone: ARC53861, RRID: AB_2862608 1:200
Anti-COL1A1 antibody ABclonal Cat#A1352, clone: N/A, RRID: AB_2760381 1:200
Anti-NLRP3 antibody ABclonal Cat#A5652, clone: N/A, RRID: AB_2766412 1:200
Anti-Caspase-1 antibody ABclonal Cat#A0964, clone: N/A, RRID: AB_2757485 1:200
Anti-IL-1β antibody ABclonal Cat#A11369, clone: N/A, RRID: AB_2758528 1:200
Anti-pIKKB antibody Cell Signaling Technology Cat#2697S, clone: 16A6, RRID: AB_2079382 1:200
Anti-IKKB antibody Cell Signaling Technology Cat#8943S, clone: N/A, RRID: AB_11024092 1:200
Anti-pNF-κb antibody Cell Signaling Technology Cat#3031S, clone: N/A, RRID: AB_330559 1:200
Anti-NF-κB antibody Cell Signaling Technology Cat#4764S, clone: C22B4, RRID: AB_823578 1:200
Anti-TRAF6 antibody ABclonal Cat#A16991, clone: N/A, RRID: AB_2772697 1:200
Anti-HIF1-α antibody Active Motif Cat#39665, clone: N/A, RRID: AB_2614934 1:200
Anti-c-MYC antibody Cell Signaling Technology Cat#13987S, clone: D3N8F, RRID: AB_2631168 1:200
Anti-pSTAT5 antibody Cell Signaling Technology Cat#4322, clone: N/A, RRID: AB_10544692 1:200
Anti-STAT5 antibody Cell Signaling Technology Cat#25656, clone: D3N2B, RRID: AB_2798908 1:200
Anti-PKM2 antibody Cell Signaling Technology Cat#4053T, clone: D78A4, RRID: AB_1904096 1:200
Anti-Ubiquitin antibody SantaCruz Cat#sc-8017, clone: P4D1, RRID: AB_628423 1μg per IP
Anti-IgG Light Chain antibody Abbkine Cat#A25012, clone: N/A, RRID: AB_2737290 1μg per IP
Anti-IgG antibody SantaCruz Cat#sc-2025, clone: N/A, RRID: AB_737182 1μg per IP
Alexa Fluor 488 goat anti-rabbit IgG Thermo Fisher Cat#A-11008, clone: N/A, RRID: AB_143165 1:400
Alexa Fluor 405 goat anti-rabbit IgG Thermo Fisher Cat#A-31556, clone: N/A, RRID: AB_221605 1:400
Alexa Fluor 647 goat anti-rabbit IgG Thermo Fisher Cat#A-21245, clone: N/A, RRID: AB_2535813 1:400
Alexa Fluor 488 goat anti-mouse IgG Jackson ImmunoResearch Cat#715-165-151, clone: N/A, RRID: AB_2315777 1:400
Alexa Fluor 594-F(ab')2 goat anti-mouse IgM+IgG (H+L) Jackson ImmunoResearch Cat#115-586-068, clone: N/A, RRID: AB_2338895 1:100
Alexa Fluor 594-F(ab')2 anti-human Ig (M+G) Jackson ImmunoResearch Cat#109-586-127, clone: N/A, RRID: AB_2337876 1:100
Biotin-F(ab')2 anti-human Ig (M+G) Jackson ImmunoResearch Cat#109-066-127, clone: N/A, RRID: AB_2337641 1:100
Biotin-conjugated F(ab')2 anti-mouse Ig (M+G) Jackson ImmunoResearch Cat#115-066-068, clone: N/A, RRID: AB_2338581 1:100
IgM specific secondary antibody Bethyl Cat#A90-101P, clone: N/A, RRID: AB_67189 1:5000
IgG1 specific secondary antibody Bethyl Cat#A90-105P, clone: N/A, RRID: AB_67150 1:5000
Anti-β-actin antibody Proteintech Cat#60008-1-IG-10, clone: 7D2C10, RRID: AB_2289225 1:1000
Anti-GAPDH antibody Proteintech Cat#60004-1-IG, clone: 1E6D9, RRID: AB_2107436 1:1000

Validation

All the antibodies used in the study were commercially available and the more information is available in the manufacturer website.

Eukaryotic cell lines

Policy information about cell lines and Sex and Gender in Research

Cell line source(s)

HC cell line and IgG4-RD cell lines were derived respectively EBV-immortalized B cells from IgG4-RD patients and healthy volunteers by Shaozhe Cai (Department of Rheumatology and Immunology, Tongji Hospital, Tongji Medical College, Huazhong University of Science and Technology). HEK293T cells were provided by Dr Hongmei Yang (Huazhong University of Science and Technology)

Authentication

Cell lines were routinely assessed by flow cytometry and vitro stimulation experiments for cell lines identity.

Mycoplasma contamination

The cells used were analysed to confirm the absence of mycoplasma contamination.

Commonly misidentified lines
(See ICLAC register)

No misidentified cell lines were used.

Animals and other research organisms

Policy information about studies involving animals; ARRIVE guidelines recommended for reporting animal research, and Sex and Gender in Research

Laboratory animals	Wild-type C57Bl/6 (C57Bl/6J, Strain #SYXK, 2021-0057) mice weighing 20-30 g (6-8 weeks old) and were purchased from the Laboratory Animal Center, Huazhong University of Science and Technology. Usp25 knockout mice were a gift from Bo Zhong (State Key Laboratory of Virology, College of Life Sciences, Wuhan University) and were 6-8 weeks old when used for the studies. Btk-floxed mice a gift from Yong-Gui Wu (Department of Nephropathy, The First Affiliated Hospital of Anhui Medical University). Btk-floxed mice were crossed with Mb1cre mice to obtain BTK knockout mice (Mb1cre+/- BTK fl/fl) and were 6-8 weeks old when used for the studies. All mice were group-housed with five per cage bred under specific-pathogen free (SPF) pathogen-specific conditions with 12 h light/12 h dark cycle at 72 "F and 40% humidty with ad libitum access to food and water. Experimental and control mice were co-housed.
Wild animals	No wild animals were used.
Reporting on sex	Animals experiments were performed with females and males and no sex determination was performed. We did not find any sex differences in any of our study, thus we combined female and male mice in all final statistical analyses.
Field-collected samples	The study did not involve samples collected from the field.
Ethics oversight	All experimental procedures with mice complied with the Chinese Council on Animal Care and approved by the Ethics Committee of the Huazhong University of Science and Technology.

Note that full information on the approval of the study protocol must also be provided in the manuscript.

Plants

Seed stocks	The study did not involve in Plants
Novel plant genotypes	The study did not involve in Plants
Authentication	The study did not involve in Plants

Flow Cytometry

Plots

Confirm that:

☒ The axis labels state the marker and fluorochrome used (e.g. CD4-FITC).

☒ The axis scales are clearly visible. Include numbers along axes only for bottom left plot of group (a 'group' is an analysis of identical markers).

☒ All plots are contour plots with outliers or pseudocolor plots.

☒ A numerical value for number of cells or percentage (with statistics) is provided.

Methodology

Sample preparation	For mouse spleen samples, the spleen was grinded and centrifuged by ficoll-Hypaque solution for 20 min to obtain mononuclear cells. For human blood samples, blood samples were collected into ethylenediamine tetraacetic acid (EDTA) tubes and processed in a biosafety level 2+ laboratory within 2 h post–blood draw. Whole blood was centrifuged at 3000rpm for 10 min at room temperature to collect the plasma. The remaining blood was diluted with PBS in a 1:1 ratio and then added into the tube of ficoll-Hypaque solution in a 1:1 ratio and centrifuged at 2000 rpm for 20 min. After centrifugation, PBMCs were collected according to different densities. Before antibody staining, Fc receptors were blocked with FcR blocking at 1:50 concentration for 10 min. For cell surface staining, PBMCs and splenic lymphocytes were stained with antibodies using optimized concentration of antibodies for 30 min at 4°C together with fixable viability Stain or 7-AAD at a concentration of 1:20. For intracellular staining, cells were fixed and permeabilized with Fixation / Permeabilization Kit before staining with antibodies. Cells were incubated on ice for 30 min for antibody staining. Following incubation, cells were washed twice with PBS. For phosphoflow cytometry, after incubation with anti-B220 or anti-CD19, cells were incubated with soluble antigen (10 µg/ml) at 4°C for 30 min. Streptavidin (20 µg/ml) were added for 10 min. Next, the cells were activated various times at 37°C and cells were fixed and permeabilized, followed by incubation with antibody. Samples were analyzed by Attune™ NxT, AFC2 (Thermo Fisher).

Instrument	Samples were analyzed by Attune™ NxT, AFC2 (Thermo Fisher).
Software	Results were analyzed by FlowJo (V10) software.
Cell population abundance	All cell populations were distinct in all experiments.
Gating strategy	Appropriate compensation controls for each fluorochromes were generated. Voltage and cell populations were set by unstained samples and stained control samples. All cells obtained were first gated on FCS/SSC (record at least 100,000 cells) and FCS/FCH to obtain single cells. Samples from the same group were recorded under the same settings. The FCS files were exported and analysed by FlowJo v10.

☒ Tick this box to confirm that a figure exemplifying the gating strategy is provided in the Supplementary Information.
